# An Account of the Efficacy of a Salivation in the Cure of the Pulmonary Consumption; in a Letter from Dr. Samuel Akerly, to Dr. Benjamin Rush

**Published:** 1808-03

**Authors:** Samuel Akerly


*An Account of the Efficacy of a Salivation in the Cure of the Pulmonary Consumption; in a Letter from Dr.* SAMUEL AKERLY, *to Dr.* BENJAMIN RUSH.

SIR,

SINCE my residence in this house, I have followed your plan in the treatment of pthisical patients, and have been more successful than with any other. The object of this letter is to strengthen your opinion, and increase the information on this subject; the way to the apt illustration of which you have pointed out.

---

My own trial with mercury in phthisis pulmonalis has not alone been successful; but accidentally meeting Dr. C. Ball, of Brooklin, Long Island, his practice corroborated mine. I am sanguine in the belief that mercury will cure a pulmonary consumption, when it has not advanced to its last stage. In those predisposed to the disease by hereditary taint or otherwise, it may be successfully given; and to effect this, gently to touch the gums is sufficient, when the cough and expectoration will cease. Mercury, as far as my trial goes, has no effect upon the hoarseness which sometimes accompanies this disease. I have generally used the mild muriate of mercury in my prescriptions, in the form of pill, accompanying it with an expectorant. The successful cases which I have noted, are inclosed, and at your disposal. I have lost several patients after they began to take calomel, but they were far gone with the disease, and despaired of, when admitted.

---

JOSEPH PURSELL, seaman, aged 20 years, was admitted into the hospital on the 8th July, 1806, for phthisis. He had been sick for some months, in which time he was very much reduced, and had become greatly debilitated. His cough was great, attended with a bloody expectoration, hoarseness and pain of the breast. His eyes were of a glossy white, and his tongue furred. In this state I prescribed calomel and a pectoral mixture, according to the following formula:

℞. Rad. serpent. Sem anisi, ana, ʒiij. Rad. glycrr. ʒij. This was boiled in ℥viij. of water, till reduced to ℥vj. When cooled and strained, a large spoonful was given every hour; this was continued with the calomel, until his mouth and gums were sore, and the evacuation by saliva saliva was considerable. By this time his expectoration decreased, and was without blood.

The above medicines were then stopped, and a solution of borate of soda was given to wash and cool the mouth. Tar pills made up with magnesia were also administered, when the calomel was stopped. With this treatment, and a milk diet, he continued to improve till the salivation ceased. Tar pills were taken till he was dismissed, which was on the 25th of the same month, having been confined for 18 days. He was requested to stay a week longer, but feeling quite strong, and eager to be discharged, he went away cured. I saw him a few weeks after, and he appeared to be getting strength and flesh.

---

J—— A—— ——, once of reputable connections, in consequence of the death of her parents, had thrown herself upon the town, and had lived several years as a prostitute, in which situation she fell into a consumption, in the nineteenth year of her age.

She was admitted on the 11th of April, 1806, with a hoarseness and severe cough, attended with a painful and difficult expectoration. Her eyes were glassy, and she had regular accessions of hectic fever in the afternoon. In this state she had lingered the preceding six months, in a house of ill fame, from whence she came to the hospital.

After her admission, she had considerable fever and pain, for which she was bled and blistered. The pectoral mixture mentioned in the preceding case was ordered, and taken for some time without much relief. Three drachms of the paregoric elixir were generally added to ℥viij of the mixture after it was prepared. It was alternated with the thebaic tincture, but neither would allay the cough. Finding the above medicine had little or no effect, it was changed for the spiritus Mindereri, which relieved the patient for a time, but her cough returned as severe as ever, when she began to despair and weep about her illness, lest she should never recover; I gave her encouragement and promised her pills, which, if she would take, I had no doubt would cure her. She was somewhat backward, however, fearing they would be mercury, the effects of which she dreaded, as some in the same ward were under a salivation. Calomel pills were, notwithstanding, given her. The quantity was small, and she continued to take three a day for upwards of a week, and still continued in the use of the spiritus Mindereri. Her cough soon abated, and had nearly ceased, when her gums began to grow sore, by which time the calomel was omitted, without producing complete ptyalism. Her legs were likewise œdematous, to which I applied bandages, and kept blisters discharging with epispastic ointment, by which means they were soon cured.

She continued to take the spiritus Mindereri, a large spoonful every hour during the day, till discharged on the 7th of June, when she went away well, and in good spirits, having been in the hospital eight weeks and a day.

---

James M'Colgan, seaman, aged 36 years, was admitted into the hospital on the 30th of May, 1806, with an inflammation of the lungs. He had an excessively dry cough, with pain of the breast and side, a full pulse, and hot skin. He was repeatedly bled, and blisters were applied to the breast. Expectorants were given him, and he appeared to be getting well, so that he walked about, and had fixed upon a day when he thought he would be able to leave the hospital. In the mean time he was taken with a violent fit of coughing, which had nearly killed him, and in which he discharged more than a quart of pure pus. After this discharge he obtained some ease, and a respite from his cough. A large quantity of the expectorant mixture and anodynes were given to allay the irritation. When the discharge ceased, a dry cough continued, upon which, after many days trial, pectoral medicines had no effect. The patient began to grow weak, and was evidently approaching to a consumption. He now despaired of getting well. In this condition I prescribed calomel in pills, in order to bring on a slow salivation. In a few days his cough was allayed, and the patient took courage, and again thought of being discharged; but before the mercury affected his mouth, his hopes were destroyed, and, for my own part, I thought he would have died.

Early one morning I was awakened by the nurse, that I might see M'Colgan. I found him in a distressed situation with a continual violent cough, pouring matter from his mouth. This continued for more than two hours, and was not allayed by powerful anodynes; the matter discharged was about two quarts.

Again finding some ease, the calomel pills were continued, and the expectorant mixture, with an increased quantity of laudanum. A slight discharge of pus by expectoration continued, which gradually decreased with the cough as the salivation came on, at which time it ceased.

ceased. His medicines were omitted, and a solution of borax, (borate of soda,) was given to cool and cleanse his mouth. This soon got well, and he went away cured on the 7th July, 1806.

---

Frederick Moline, seaman, by birth a Swede, aged 39 years, was admitted into the hospital on the 10th October, 1806, with phthisis. He complained of pain in the breast, frequent cough, and a bad tasted expectoration. His eyes were glassy, his tongue foul, and his pulse weak. His countenance wore the aspect of despondency. Having been sick three months, and unable to work, with a family to support, his complaint increasing upon him, he despaired of getting well. Thus circumstanced, he was received into the hospital. From my firm belief in the good effects of mercury, I immediately prescribed it with the mixture before mentioned. When his gums were affected, the cough and expectoration abated; the calomel was omitted, having produced only a slight ptyalism, but the expectorant was continued. The pain in the breast was removed by a blister, but it returned in the form of rheumatism about the shoulders; another blister relieved, but did not remove it. Tincture of capsicum was then applied externally with some advantage. The patient left the hospital on the 5th of November following, cured of his pulmonary complaint, though still feeling a slight pain about the shoulders, but his countenance was enlivened, his pulse raised, and his health apparently established.

